# Reduced risk of heart failure with intensified multifactorial intervention in individuals with type 2 diabetes and microalbuminuria: 21 years of follow-up in the randomised Steno-2 study

**DOI:** 10.1007/s00125-018-4642-y

**Published:** 2018-05-30

**Authors:** Jens Oellgaard, Peter Gæde, Peter Rossing, Rasmus Rørth, Lars Køber, Hans-Henrik Parving, Oluf Pedersen

**Affiliations:** 1 Department of Cardiology and Endocrinology, Slagelse Hospital, Slagelse, Denmark; 2 Institute for Regional Health Research, University of Southern Denmark, Odense, Denmark; 3 Steno Diabetes Center Copenhagen, Gentofte, Denmark; 4 Faculty of Health, University of Aarhus, Aarhus, Denmark; 5 Faculty of Health, University of Copenhagen, Copenhagen, Denmark; 6 Department of Cardiology, Rigshospitalet, Copenhagen, Denmark; 7 Department of Endocrinology, Rigshospitalet, Copenhagen, Denmark; 8 Novo Nordisk Foundation Center for Basic Metabolic Research, University of Copenhagen, Blegdamsvej 3B, DK-2100 Kbh Ø, Denmark

**Keywords:** Complications, Heart failure, Microalbuminuria, Multifactorial intervention, NT-proBNP, Type 2 diabetes

## Abstract

**Aims/hypothesis:**

In type 2 diabetes mellitus, heart failure is a frequent, potentially fatal and often forgotten complication. Glucose-lowering agents and adjuvant therapies modify the risk of heart failure. We recently reported that 7.8 years of intensified compared with conventional multifactorial intervention in individuals with type 2 diabetes and microalbuminuria in the Steno-2 study reduced the risk of cardiovascular disease and prolonged life over 21.2 years of follow-up. In this post hoc analysis, we examine the impact of intensified multifactorial intervention on the risk of hospitalisation for heart failure.

**Methods:**

One hundred and sixty individuals were randomised to conventional or intensified multifactorial intervention, using sealed envelopes. The trial was conducted using the Prospective, Randomised, Open, Blinded Endpoints (PROBE) design. After 7.8 years, all individuals were offered intensified therapy and the study continued as an observational follow-up study for an additional 13.4 years. Heart-failure hospitalisations were adjudicated from patient records by an external expert committee blinded for treatment allocation. Event rates were compared using a Cox regression model adjusted for age and sex.

**Results:**

Eighty patients were assigned to each treatment group. Ten patients undergoing intensive therapy vs 24 undergoing conventional therapy were hospitalised for heart failure during follow-up. The HR (95% CI) was 0.30 (0.14, 0.64), *p* = 0.002 in the intensive-therapy group compared with the conventional-therapy group. Including death in the endpoint did not lead to an alternate overall outcome; HR 0.51 (0.34, 0.76), *p* = 0.001. In a pooled cohort analysis, an increase in plasma N-terminal pro-B-type natriuretic peptide (NT-proBNP) during the first two years of the trial was associated with incident heart failure.

**Conclusions/interpretation:**

Intensified, multifactorial intervention for 7.8 years in type 2 diabetic individuals with microalbuminuria reduced the risk of hospitalisation for heart failure by 70% during a total of 21.2 years of observation.

**Trial registration:**

ClinicalTrials.gov NCT00320008.

**Electronic supplementary material:**

The online version of this article (10.1007/s00125-018-4642-y) contains peer-reviewed but unedited supplementary material, which is available to authorised users.

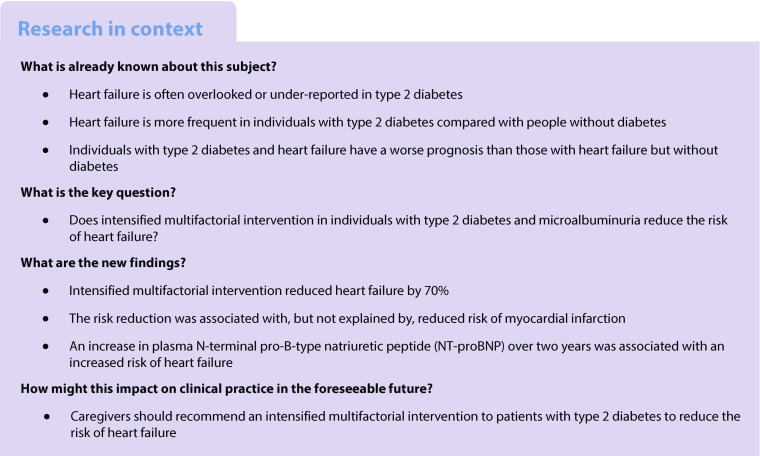



## Introduction

Individuals with type 2 diabetes mellitus are at a high risk of developing congestive heart failure, having a relative risk at least twice as high as individuals without diabetes, especially when urinary albumin excretion rate (u-AER) is elevated [1]. Heart failure is a serious complication in type 2 diabetes, with a median survival from diagnosis of 3.5 years [2] and a 5-year mortality rate of 75% [3]. The prognosis of individuals with type 2 diabetes and heart failure is worse than that of heart-failure patients without diabetes mellitus [4].

Despite the increased risk and unfavourable prognosis, heart failure in type 2 diabetes traditionally has been sparsely reported in cardiovascular outcomes trials for glucose-lowering drugs [5, 6]. Recent trials, however, have an increased focus on heart failure, but with no consensus between studies or specific guidance from regulatory bodies [7, 8] in the methodology used to define prevalent and incident heart failure, increasing the risk of bias.

Congestive heart failure in diabetes mellitus may be divided into a primary form termed metabolic or diabetic cardiomyopathy, and a secondary form predominantly caused by coronary ischaemia [9–12]; however, distinct definitions that can be used to differentiate do not exist. Causal factors for the development of primary heart failure include hypertension, fluid overload and possibly substrate overload, causing accumulation of intracellular fat and subsequent reduced contractility of cardiomyocytes [12].

In the Steno-2 study, we compared conventional multifactorial management of patients with type 2 diabetes and microalbuminuria (24 h urinary albumin excretion 30–300 mg) with intensified multifactorial intervention targeting known modifiable risk factors with individualised lifestyle intervention and tailored polypharmacy [13–15] at a specialised diabetes clinic. After 3.8 years of intervention, patients who received intensified treatment had a reduction in the hazard of microvascular complications of around 50% [13]; after 7.8 years of intervention, a 53% reduction was seen in cardiovascular endpoints in the arm that was allocated to intensified intervention [14]. At this point, the formal randomisation was neutralised and all patients were offered intensified treatment as in the original intensive-therapy arm and the trial continued as an observational follow-up study. Five years after the end of the trial (in total 13.3 years of follow-up), we reported a 46% reduction in total mortality of patients in the arm originally allocated the intensive intervention [15]. We have also recently, in a 21.2-year follow-up after trial initiation, demonstrated that the intensified multifactorial approach increased life length with a median of 7.9 years, a life gain that was matched by time free of incident ischaemic heart disease of 8.1 years [16]. During the same period, progression in nephropathy and loss of kidney function was diminished, which led to a reduction in the risk of end-stage renal disease that did not fulfil the pre-specified statistical significance definition, however (*p* = 0.061) [17].

In the present post hoc analysis, we report the 21.2-year risk of developing heart failure in patients with type 2 diabetes and microalbuminuria, who were allocated conventional multifactorial care or intensified multifactorial care for a trial period of 7.8 years.

## Methods

The detailed protocol for patient inclusion, randomisation and intervention, as well as for anthropometric, clinical and biochemical measurements, has been reported previously [13]. The protocol for the follow-up trial was in accordance with the declaration of Helsinki and approved by the local ethics committee (Ethics Committee, Capital Region of Denmark; protocol ID number: H-KA-99035-GS, add. 41104) and by the Danish Data Protection Agency (J.Nr. 2015-41-4042) and the trial was registered at ClinicalTrials.gov, number NCT00320008. All participants gave their informed consent upon randomisation and confirmed that on follow-up visits.

### Patients

**Fig. 1:**
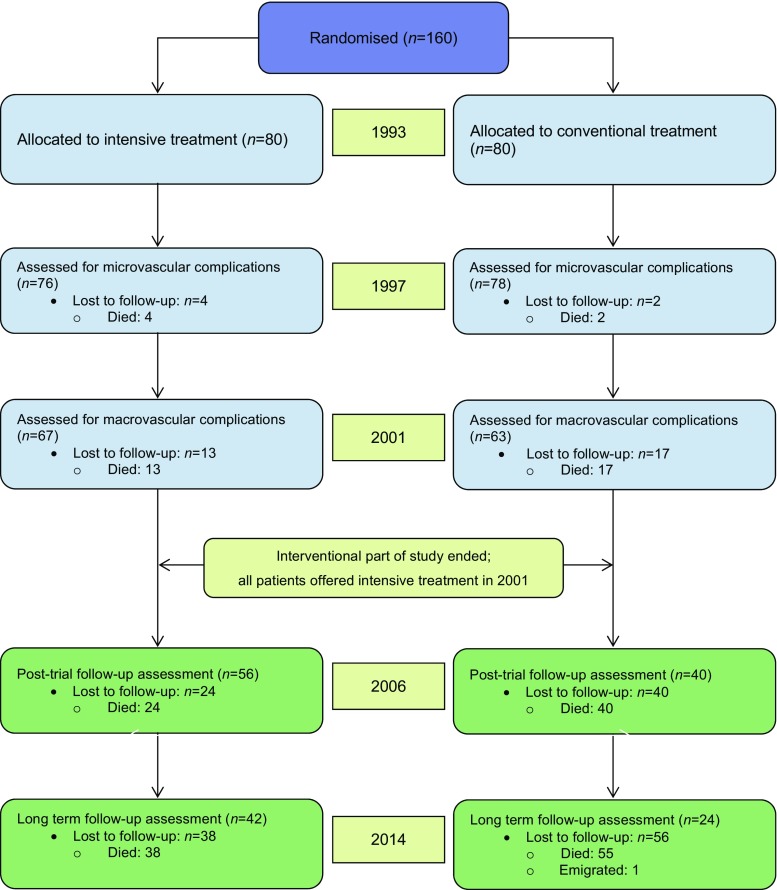
CONSORT diagram showing patient flow throughout the trial. The first 7.8 years were the active intervention period, after which time the randomisation was neutralised and continued as a post-trial observational follow-up study with all remaining patients being offered the same treatment as in the original intensive-therapy group

One hundred and sixty Danish patients with type 2 diabetes and microalbuminuria were randomised from year 1993, using sealed envelopes, to either conventional or intensified multifactorial intervention. Individuals were followed for up to 21.2 years (Fig. 1). The trial was conducted using the Prospective, Randomised, Open, Blinded Endpoints (PROBE) design.

### Intervention

The treatment regimen in both randomisation groups was target driven, with targets described in Table 1. In the conventional arm, targets followed national recommendations at all times. The intensive arm had stricter glycaemic, lipid and blood pressure targets, and received ACE inhibitors and aspirin therapy. In order to achieve targets, means of regulation were applied in a sequential manner starting with lifestyle modification including weight loss, smoking cessation and increased physical activity with subsequent addition of whichever pharmacological agent(s) were needed to achieve the specific targets.

**Table 1:** Treatment targets for treatment groups

	Intensive		Conventional	
	1993–1999	2000–2001	1993–1999	2000–2001
Systolic blood pressure (mmHg)	<140	<130	<160	<135
Diastolic blood pressure (mmHg)	<85	<80	<95	<85
HbA_1c_ (%)	<6.5	<6.5	<7.5	<6.5
HbA_1c_ (mmol/mol)	<48	<48	<58	<48
Fasting serum total cholesterol (mmol/l)	<4.9	<4.5	<6.5	<4.9
Fasting serum triacylglycerol (mmol/l)	<1.7	<1.7	<2.2	<2.0
Treatment with ACE inhibitor irrespective of BP	Yes	Yes	No	Yes
Aspirin therapy				
Known ischaemia	Yes	Yes	Yes	Yes
Peripheral vascular disease	Yes	Yes	No	No
No known vascular disease	No	Yes	No	No

The conventional-therapy group was at all times treated with targets as least as strict as recommended in national guidelines. Aspirin treatment was initiated if any of the indication criteria were met. The mean intervention duration was 7.8 years and thereafter all patients were offered treatment similar to that of the original intensive-therapy group

### Endpoint definitions and data

The primary endpoint of this current follow-up study was hospitalisation with congestive heart failure. The secondary endpoints were time-to-first-event of composites of heart failure or cardiovascular death and of heart failure or all-cause death. Individuals were followed by in-trial study visits after approximately 2, 4, 8, 13 and 21 years after randomisation (Fig. 1). At these visits, patients had a comprehensive screening for micro- and macrovascular complications performed as described in [16].

Heart failure was defined as described in electronic supplementary material (ESM) Methods, including prespecified criteria for symptoms, signs and treatment initiated or intensified. Outcome data were extracted from patient records and adjudicated by an external expert committee that was blinded to treatment allocation. Mortality data were collected from the Danish Civil Registry.

Baseline ejection fraction (EF) was calculated using the modified Quinones formula [18]. We were not able to correct for apical movement, resulting in a likely overestimation of baseline EF. No individuals had, however, any symptoms of heart failure at baseline, thus we assume the effect to be equal in the two treatment groups of patients.

### Statistical methods

Baseline characteristics were compared using a *t* test for data with Gaussian distribution and the Mann–Whitney *U* test for data with a non-Gaussian distribution. Time-to-event analyses were conducted using a Cox proportional hazards model with treatment as a covariate and illustrated using cumulative incidence function (CIF) curves. The primary analyses were adjusted for age and sex. Furthermore, a model adjusted for BMI, plasma N-terminal pro-B-type natriuretic peptide (NT-proBNP), EF, systolic blood pressure, HbA_1c_, u-AER and GFR measured by ^51^Cr-EDTA clearance (corrected for body surface area [19]) was applied with a stepwise backward elimination approach, with a threshold for staying in the model of *α* = 0.10. Proportional hazards were checked by visual inspection of CIF curves. Stepwise backward elimination was chosen to reduce the risk of overfitting the model. We performed the Cox regressions without elimination of variables as sensitivity analyses.

A number of exploratory analyses were carried out to confirm the validity of the results: in order to investigate the difference in distribution of primary and secondary heart-failure events between groups, and to evaluate the impact of interventions on each type, we used a competing-risks regression model (Fine and Gray [20]) to assess competing risk from myocardial infarction (MI) and from death in an analysis where death was not included in the endpoint. We considered the cases where MI occurred before a hospitalisation for heart failure as secondary heart failure. Estimates were, in these analyses, reported as a sub-distribution hazard ratio (SHR) with 95% CIs. In addition, we performed sensitivity analyses for the primary and secondary outcomes excluding baseline EF from the further-adjusted Cox regressions of primary and secondary outcomes. These analyses were carried out because of the observation that baseline EF was missing for 15 individuals (9.4%) (five in the intensive-therapy group; ten in the conventional-therapy group). We also investigated whether the change in plasma NT-proBNP and u-AER values during the first two years of intervention was associated with the primary and secondary outcomes by adding the delta value divided into tertiles to the further-adjusted Cox regression.

Numerical results are presented followed by 95% CIs in brackets. Significance level was set at *α* = 0.05. Statistical analyses were performed using STATA/IC version 15 (StataCorp, College Station, TX, USA).

## Results

Baseline patient characteristics are presented in Table 2. Individuals in the two groups were similar with regards to anthropometrics, and to clinical and biochemical measures. No individuals had symptoms or clinical signs (including echocardiographic evidence) of heart failure at baseline. Mean levels of plasma NT-proBNP were in the upper range of the normal interval. All patients had microalbuminuria, but preserved renal function.

**Table 2:** Baseline clinical, anthropometric and biochemical data

	Baseline 1993	
Clinical variable (mean ± SD)	Intensive (*N* = 80)	Conventional (*N* = 80)
Age (years)	54.9 ± 7.2	55.2 ± 7.2
Proportion males (%)	79	70
Diabetes duration (years) median (range)	4 (0; 30)	6 (0; 29)
Systolic BP (mmHg)	146 ± 11	149 ± 19
HbA_1c_ – (mmol/mol) HbA_1c_ – (%)	68 ± 6 8.4 ± 2.7	73 ± 5 8.8 ± 2.6
BMI (kg/m^2^) (SD)	29.7 (3.8)	29.9 (4.9)
GFR (ml/min/1.73m^2^)	116 ± 24	118 ± 25
u-AER (mg/24 h) median (IQR)	78 (61; 120)	69 (47; 113)
Plasma NT-proBNP (pmol/l) median (IQR)	35 (12; 71)^a^	32 (13; 67)
Plasma NT-proBNP ≥ 100 pmol/l (N [%])	14 (18)^a^	16 (20)
Left ventricle EF (SD)	67 (8)^b^	67 (8)^c^

^a^One patient with missing data

^b^Five patients (9%) with missing data

^c^Ten patients (12.5%) with missing data

IQR, interquartile range

Individuals were followed for up to 21.9 years after baseline with mean follow-up of 21.2 years for those surviving to the end of follow-up.

### Primary and secondary outcomes

#### Primary outcome

**Fig. 2:**
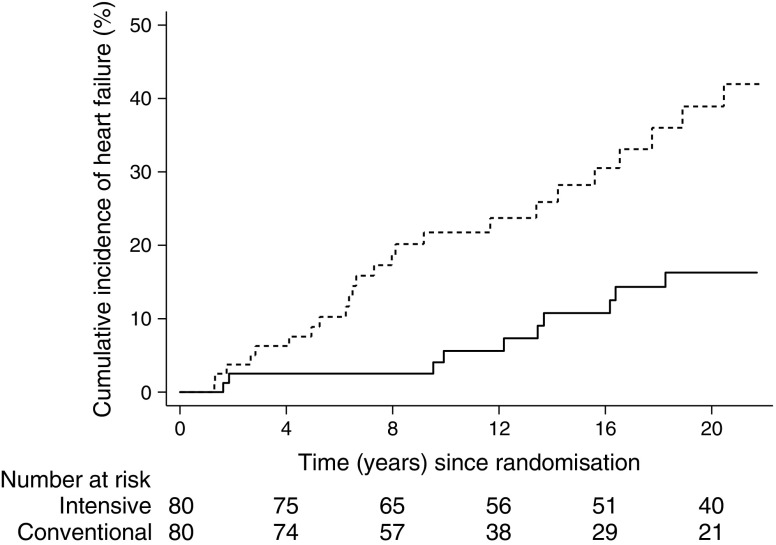
CIF plot of hospitalisation for heart failure. Dashed line, conventional therapy; solid line, intensive therapy. The unadjusted relative hazard reduction was 69% in the intensive-therapy group. Logrank *p* = 0.001

Over the entire study course, ten patients (13%) in intensive-therapy group developed heart failure vs 24 patients (30%) in the conventional-therapy group. All events complied with the formal definition of the primary outcome. The age- and sex-adjusted HR was 0.30 (0.14, 0.64), *p* = 0.002 in the intensive-therapy group compared with the conventional-therapy group (Fig. 2). The further-adjusted (BMI, HbA_1c_, u-AER, GFR, EF, systolic blood pressure and NT-proBNP) HR was 0.23 (0.10, 0.54), *p* = 0.001 with age (*p* = 0.017), BMI (*p* = 0.010) baseline EF (*p* = 0.032) staying in the final model as independent covariates along with treatment allocation. Baseline GFR (*p* = 0.065), u-AER (*p* = 0.070) and HbA_1c_ (*p* = 0.077) were all of borderline significance.

#### Secondary outcome

**Fig. 3:**
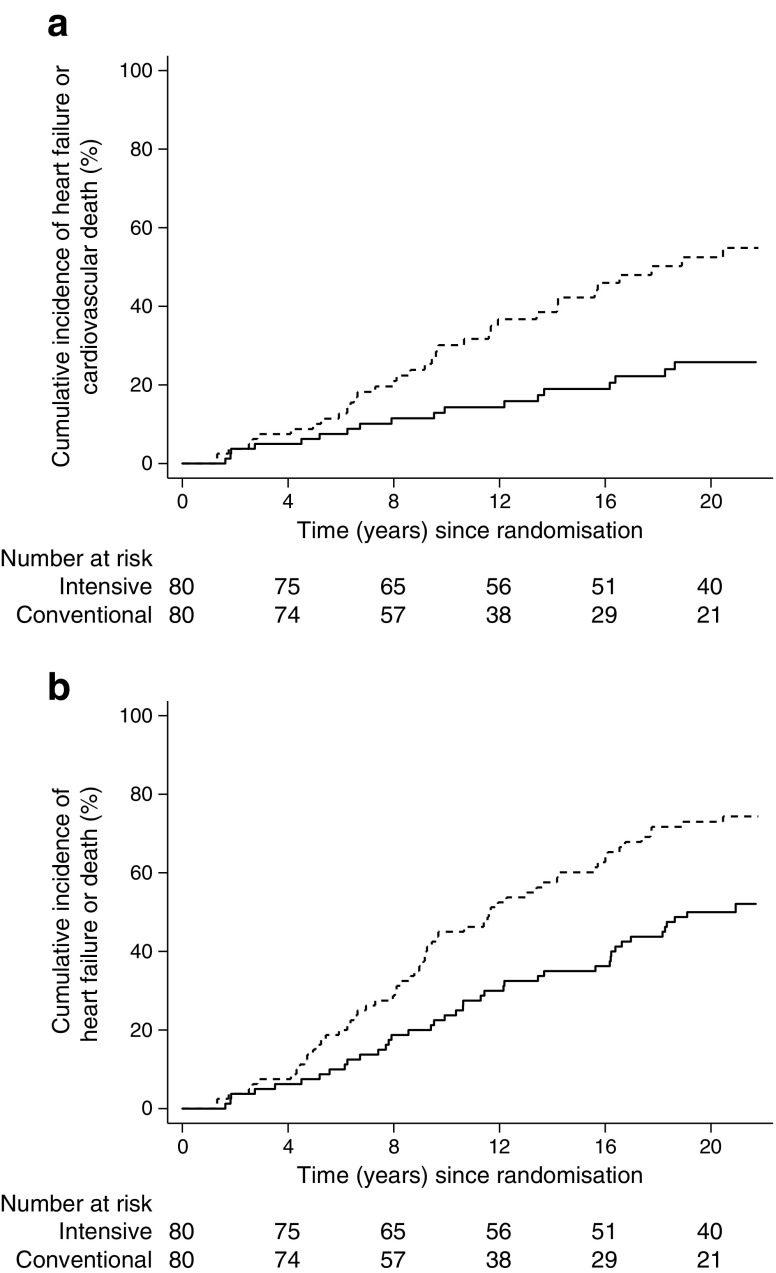
CIF plots of the secondary outcomes. Dashed line, conventional therapy; solid line, intensive therapy. (**a**) Heart failure or cardiovascular death. The unadjusted relative hazard reduction was 61% in the intensive-therapy group. Logrank *p* < 0.001. (**b**) Heart failure or death from all causes. The unadjusted relative hazard reduction was 48% in the intensive-therapy group. Logrank *p* = 0.001

The secondary outcomes are illustrated in Fig. 3. Eighteen patients (23%) in the intensive-therapy group and 35 patients (44%) in the conventional-therapy group reached the secondary endpoint of heart-failure hospitalisation or death from CVD, leading to an age- and sex-adjusted HR of 0.38 (0.22, 0.68), *p* = 0.001. Further adjusted, the HR was 0.31 (0.16, 0.58), *p* = 0.001. Age (*p* = 0.001), BMI (*p* = 0.023) and baseline HbA_1c_ (*p* = 0.011), EF (*p* = 0.003) and GFR (*p* = 0.011) stayed in the model after elimination and u-AER (*p* = 0.084) was of borderline significance.

Forty-one patients (51%) originally allocated to intensified therapy and 59 patients (74%) allocated to conventional therapy reached the combined endpoint of heart failure or death from all causes. The HR was 0.51 (0.34, 0.76), *p* = 0.001. This estimate was stable in the further-adjusted model, with age (*p* < 0.001), GFR (*p* = 0.050) and plasma NT-proBNP (*p* = 0.033) staying in the model in addition to treatment allocation, and with EF (*p* = 0.074) and HbA_1c_ (*p* = 0.090) being of borderline significance.

### Exploratory analyses

#### Competing risks from MI

**Fig. 4:**
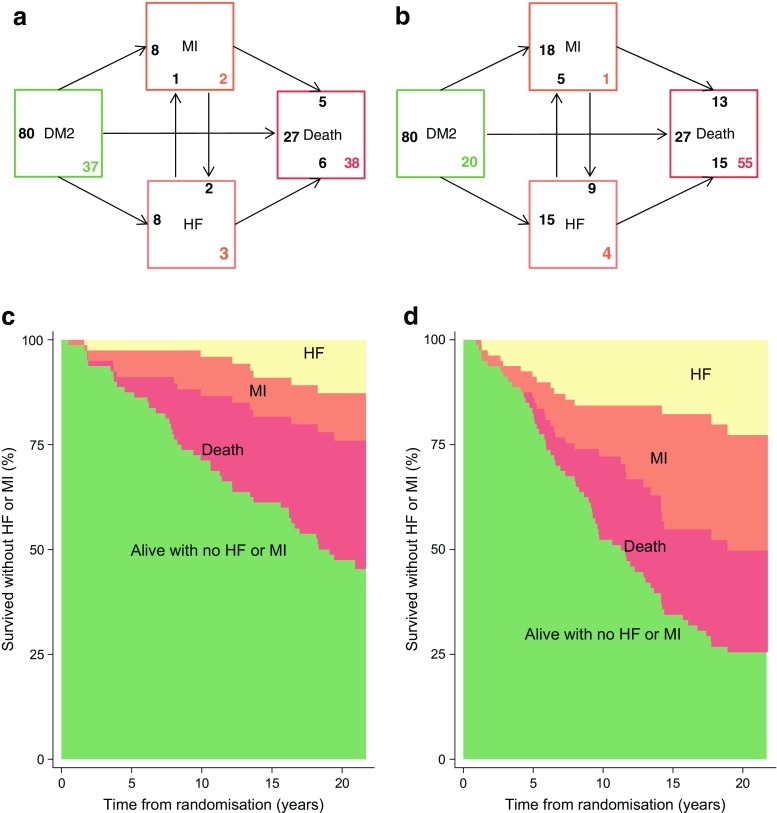
Transition frequencies from entry to MI, HF and/or death. (**a**) Intensive-therapy group and (**b**) conventional-therapy group: arrows terminate at the event and originate from the original state of the patients. The black number at the arrow end is the number with the given event coming from the state at arrow origin. The coloured number in the bottom right corner is the number of patients not progressing from the given state. Example: ten intensive-therapy patients developed HF (pale orange box, Fig. 4a). Eight had no previous MI; two developed HF after previous MI. One developed MI after HF, six died after HF and three ended the observation alive with HF. Twenty-seven patients died without prior MI or HF. In the primary analysis of data from the 21.2 years of follow-up, 26 patients in the intensive-therapy and 29 in the conventional-therapy group were classified as having died from non-CV causes and 12 vs 26 patients died from CV causes, respectively. Thirty-seven patients in the intensive-therapy group and 20 patients in the conventional-therapy group ended follow-up alive and with no incident HF or MI during follow-up. (**c**) Intensive-therapy group and (**d**) conventional-therapy group: survival frequencies without MI/HF. Both MI and HF were more frequent in the conventional-therapy group (**b**) and the difference in HF was not driven primarily by increased MI risk. DM2, type 2 diabetes mellitus; HF, heart failure

In total, nine individuals in the intensive-therapy group and 23 in the conventional-therapy group experienced an MI during the study. Of those, two in the intensive-therapy group and nine in the conventional-therapy group experienced MI *prior* to hospitalisation for heart failure (Figs 4a, b). The SHR of heart failure controlled for MI in the competing-risks regression was 0.37 (0.17, 0.78), *p* = 0.009, meaning that the observed difference in hospitalisation for heart failure was not explained by the difference in prior MI between groups. A competing-risks regression using all-cause death as the competing event to heart failure also confirmed the result with SHR of 0.36, *p* = 0.008.

Among individuals with incident heart failure, seven (70%) in the intensive-therapy group and 20 (83%) in the conventional-therapy group died during follow-up (Fig. 4). Median time from hospitalisation for heart failure to death was 2.9 years and did not differ between the two treatment groups. For MI, the corresponding numbers were six (67%) patients in the intensive-therapy and 18 (75%) in the conventional-therapy group with median time from MI to death being 2.1 years (please note that these numbers differ from the numbers in Figs 4a, b because there, patients may progress from, e.g., MI to heart failure and then die subsequently, which is not possible to control for in the simple analysis described above).

#### Sensitivity analyses excluding EF in the Cox regression

Estimates of HR for the primary and the secondary endpoints were stable when removing baseline EF from the model (ESM Table 1). Using Cox regression without elimination of variables confirmed the results, without any change of HR estimates (ESM Table 2).

The inclusion of plasma NT-proBNP as a dichotomous variable with a cut-off of 100 pmol/l in the further-adjusted model instead of as a continuous variable showed a significant association with heart failure (HR 3.2 for high vs low NT-proBNP [*p* = 0.09]) and with heart failure plus all-cause death (HR for high vs low NT-proBNP 1.9 [*p* = 0.015]).

#### Change in plasma NT-proBNP over the first two years of follow-up

Change in plasma NT-proBNP during the first two years was significantly associated with the primary outcome (in the period from year 2 to 21) in the adjusted Cox regression model. Individuals (pooled cohort) with changes in the highest tertile of delta plasma NT-proBNP (i.e. the largest increase) had a 2.7-fold ([1.19, 5.93], *p* = 0.018) increased risk of heart failure as compared with individuals in the lower two tertiles of change. Individuals (pooled cohort) in the second tertile did not differ significantly in risk from those in the lowest tertile (*p* = 0.14). Assessing an interaction between change in NT-proBNP and treatment allocation revealed that this finding was driven by the conventional-therapy group. In the conventional-therapy group, individuals with changes in the higher tertile had a threefold increased risk of heart failure compared with those in the two lower tertiles (*p* = 0.030), whereas this was not the case for the intensive-therapy group (*p* = 0.60). Change in plasma NT-proBNP was not associated with cardiovascular mortality combined with heart failure, but had a positive correlation with all-cause mortality; HR = 1.86 (1.18, 2.92), *p* = 0.07 for the highest tertile compared with the lower two tertiles. These findings were not affected by treatment allocation, but significantly more patients in the conventionally treated therapy group were in the upper tertile of plasma NT-proBNP (32 vs 19; *p* = 0.041).

In similar analyses, change in albuminuria was not associated with any of the outcomes (ESM Table 3).

## Discussion

Heart failure is a major health issue and a risk factor for early death and disability in type 2 diabetes. Until now, no trials of intensified multifactorial intervention in type 2 diabetes have investigated the potential beneficial effects of this treatment modality on congestive heart failure. In this post hoc analysis of outcome data from the Steno-2 randomised trial, we demonstrate that intensified multifactorial treatment in patients with type 2 diabetes mellitus, microalbuminuria and preserved baseline EF for 7.8 years reduced the hazard of developing clinical heart failure by 70% at 21.2 years of follow-up.

Twenty per cent of all patients developed heart failure, equalling the number of patients experiencing MI (20%), stroke (22%), amputation (19%) or blindness (19%) [16] and mortality seemed to be at least as high for patients with incident heart failure as for patients experiencing an MI. We also found that the reduction in heart failure was influenced by, but not dependent on, reduction of ischaemic heart disease risk. The results were unchanged over multiple sensitivity analyses including testing for common confounders and competing risk. Our results clearly underline the importance of applying an intensive, multifactorial approach to the management of type 2 diabetes.

In the following studies (all including patients with type 2 diabetes and micro- or macroalbuminuria), heart failure was more common than other cardiovascular complications: reduction of Endpoints in NIDDM with the Angiotensin II Antagonist Losartan (RENAAL) [21], the Irbesartan Diabetic Nephropathy Trial (IDNT) [22], the Aliskiren Trial in Type 2 Diabetes Using Cardiorenal Endpoints (ALTITUDE) [23] and Microvascular outcomes in the Heart Outcomes Prevention Evaluation Study (MICRO-HOPE) [24]. However, the Steno-2 study had significantly longer follow-up, thus allowing more time for atherosclerosis progression. It is unlikely that the risk reductions seen in the Steno-2 study are attributable to one single component of the multifactorial treatment regimen, but rather to the combination of polypharmacy and lifestyle intervention. The drug pattern was highly complex (ESM Figs 1, 2), but the use of ACE inhibitors, angiotensin II receptor blockers (ARBs), statins and aspirin was more frequent in the intensive-therapy group, which is probably reflected in our findings. In particular, we would anticipate the effect of more frequent use of ACE inhibitors and ARBs to be prominent.

Our findings suggest that diabetes caregivers should pay attention to early signs and symptoms of congestive heart failure including left-ventricular dysfunction and, together with cardiologists, take appropriate actions. Measuring plasma NT-proBNP might be helpful in assessing and monitoring risk of heart failure since high values or large increments were strongly associated with heart-failure outcomes and could be used to guide diabetologists to refer patients for evaluation by cardiologists. Our findings indicate that the higher risk of heart failure associated with an increase in NT-proBNP can be mitigated by intensified multifactorial intervention. However, evidence that intensive lifestyle improvement may increase NT-proBNP and the fact that NT-proBNP levels are inversely related to BMI warrants caution when interpreting our findings regarding NT-proBNP [25, 26].

Glucose-lowering drugs, as well as adjuvant therapies for diabetes comorbidities, modify the risk of developing heart failure. In 2009, increased risk of heart failure in diabetic patients treated with rosiglitazone in the Rosiglitazone Evaluated for Cardiac Outcomes and Regulation of Glycaemia in Diabetes (RECORD) trial [27] was reported and subsequently, the use of dipeptidyl peptidase 4 (DPP-4) inhibitors also has been flagged as potentially increasing the risk of heart failure [28]. Although the latter findings have been questioned [29, 30], the Food and Drug Administration (FDA) recently added heart-failure warnings to the Summary of medicinal Products Characteristics (SmPC) for all marketed DPP-4-inhibitors [31]. In contrast, diabetes trials with the sodium–glucose cotransporter 2 (SGLT-2) inhibitors empagliflozin and canagliflozin have demonstrated heart-failure risk reduction [32–34], whereas diabetes treatment with GLP-1 receptor agonists seem to have a neutral effect on the risk of heart failure [35–37]. In addition, the extensive use of ACE inhibitors [24, 38], ARBs, beta blockers and statins [39] in type 2 diabetes may modify the risk of heart failure, although the evidence regarding the latter is weak.

This post hoc analysis has limitations: the Steno-2 study has a small sample size of 160 individuals and the type 2 diabetes study population with microalbuminuria is selected as a high-risk patient group. Therefore, the magnitude of risk reduction we demonstrate here might not be attributable to a population of lower risk. Furthermore, the sample size leads to an inherent risk of committing type 1 statistical errors due to unaccountable bias. The fact that the results presented in this paper are in accordance with previously published results regarding other manifestations of cardiovascular disease can be regarded both as a strength and as a weakness, the latter underlined by the above statement of unmeasurable bias driving the difference in outcomes between groups.

We do not have complete echocardiographic data available for the entire patient population and baseline EF was based on a calculated estimate. Therefore, whether individuals had preserved or reduced EF prior to and after hospitalisation is uncertain. However, recent evidence shows that the prognosis is independent of EF [3].

In the Steno-2 trial and during trial follow-up, the use of sulfonylurea drugs was frequent and even between groups (ESM Fig. 1) and the use of ACE inhibitors and beta blockers was more frequent in the intensive-therapy group (ESM Fig. 2), but the total exposure to glitazones, DPP-4 inhibitors, GLP-1 inhibitors and SGLT-2 inhibitors etc. was very limited (all <5% of patients in each group at each follow-up point). The effects on heart failure of the above-mentioned drugs that have been seen in recent large-scale clinical trials of cardiovascular outcomes occur on top of the standard of care; in most current recommendations, the standard of care resembles the treatment targets for the patients originally allocated to intensified multifactorial care in the Steno-2 trial.

The significant and meaningful risk reductions reported here should be seen as a benefit of intervention against traditional risk factors; the introduction of novel glucose-lowering drugs with pleiotropic effects, on top of improved glucose control, that reduce the risk of cardiovascular morbidity and mortality by other, not presently fully elucidated, mechanisms should lead to optimism in the field of diabetes care.

In conclusion, our study demonstrated hospitalisation for heart failure to be a frequent and fatal complication in patients with type 2 diabetes and microalbuminuria. Intensified multifactorial intervention significantly reduced the occurrence of this outcome.

## Electronic supplementary material

ESM (PDF 154 kb)

## Data Availability

Data is available upon reasonable request to the corresponding author.

## Abbreviations

ARB: Angiotensin II receptor blocker
CIF: Cumulative incidence function
CVD: Cardiovascular disease
DPP-4: Dipeptidyl peptidase 4
EF: Ejection fraction
GLP-1: Glucagon-like peptide 1
MI: Myocardial infarction
NT-proBNP: N-terminal pro-B-type natriuretic peptide
SGLT-2: Sodium–glucose cotransporter 2
SHR: Sub-distribution hazard ratio
u-AER: Urinary albumin excretion rate
