# Supplementary material for: Reduced risk of heart failure with intensified multifactorial intervention in individuals with type 2 diabetes and microalbuminuria: 21 years of follow-up in the randomised Steno-2 study
Source: Diabetologia. 2018 May 30;61(8):1724–33. doi: 10.1007/s00125-018-4642-y (PMC6061176; doi:10.1007/s00125-018-4642-y)
Supplement: Supplementary file 1 — (PDF 154 kb) [file 125_2018_4642_MOESM1_ESM.pdf]

Supplementary materials

ESM Methods: Heart failure outcome definition

ESM Results: Additional results from exploratory analyses

ESM Figures: Patient-reported drug use at follow-up points during trial

ESM Methods

Outcome definitions

A. Hospitalization for Heart Failure (HF)

Presentation to an acute care facility and requiring an overnight hospitalization (change in calendar day) with an newly diagnosed or exacerbation of heart failure requiring treatment meeting the following criteria:

1. Symptoms and signs of heart failure:

One or more of the following symptoms consistent with heart failure:

a) Worsening dyspnea

b) Worsening orthopnea

c) Paroxysmal nocturnal dyspnea

d) Increasing fatigue/ worsening exercise tolerance

AND

Two or more of the following signs consistent with heart failure:

a) Rapid weight gain

b) Pulmonary edema or rales

c) Elevated jugular venous pressure

d) Radiologic signs of heart failure

e) Peripheral edema

f) Increasing abdominal distension or ascites

g) S_3 gallop

h) Hepatojugular reflux

i) Elevated brain natriuretic peptide (BNP) or N-terminal proBNP (> Upper Reference Limit)

<u>AND</u>

2. Treatment

Treatment with intravenous diuretics, intravenous vasodilators, intravenous inotropes, mechanical fluid removal (e.g., ultrafiltration or dialysis), or insertion of an intra-aortic balloon pump for hemodynamic compromise. Initiation of standing oral diuretics or intensification (doubling) of the maintenance diuretic dose will also qualify.

ESM results

In the following section, results from the exploratory analyses are presented.

ESM table 1: Results of the primary and the secondary endpoints by Cox-regression analyses omitting baseline ejection fraction from the model.

**Heart failure**	Hazard Ratio	[95% Conf. Lower bound	Interval] Upper bound	P-value
Intensive therapy	0.264	0.126	0.597	0.001
Age	1.089	1.027	1.145	0.003
NT-proBNP baseline	1.002	1.001	1.003	0.003
BMI baseline	1.080	1.006	1.160	0.034
**Heart failure / CV death**	Hazard Ratio	[95% Conf. Lower bound	Interval] Upper bound	P-value
Intensive therapy	0.364	0.201	0.659	0.001
Age	1.081	1.030	1.135	0.002
GFR baseline	0.989	0.976	1.002	0.096
NT-proBNP baseline	1.002	1.001	1.003	0.000
HbA_{1c} baseline	1.018	1.003	1.033	0.160
BMI baseline	1.061	1.000	1.127	0.050
**Heart failure / all cause death**	Hazard Ratio	[95% Conf. Lower bound	Interval] Upper bound	P-value
Intensive therapy	0.510	0.338	0.765	0.001
Age	1.080	1.044	1.117	0.000
GFR baseline	0.990	0.981	0.999	0.035
NT-proBNP baseline	1.002	1.001	1.003	0.001
HbA_{1c} baseline	1.010	0.999	1.020	0.077

BMI: Body Mass Index; GFR: Glomerular filtration rate.

ESM table 2: Results of the primary and the secondary endpoints by Cox-regression analyses without elimination of variables

**Heart failure**	Hazard Ratio	[95% Conf. Lower bound	Interval] Upper bound	P-value
Intensive therapy	0.219	0.089	0.539	0.001
Age	1.099	1.018	1.188	0.016
Sex	1.013	0.385	2.664	0.980
Ejection fraction baseline	0.954	0.908	1.002	0.061
NT-proBNP baseline	1.001	1.000	1.003	0.103
HbA_{1c} baseline	1.020	0.999	1.041	0.066
u-AER baseline	1.006	1.000	1.012	0.063
GFR baseline	0.991	0.973	1.009	0.321
Systolic BP baseline	1.000	0.977	1.023	0.977
Total cholesterol baseline	1.028	0.739	1.429	0.872
BMI baseline	1.102	1.010	1.202	0.028

**Heart failure / CV death**	Hazard Ratio	[95% Conf. Lower bound	Interval] Upper bound	P-value
Intensive therapy	0.273	0.137	0.543	0.000
Age	1.105	1.040	1.175	0.001
Sex	1.104	0.501	2.434	0.806
Ejection fraction baseline	0.946	0.910	0.984	0.006
NT-proBNP baseline	1.001	1.000	1.003	0.151
HbA_{1c} baseline	1.023	1.007	1.040	0.006
u-AER baseline	1.004	0.999	1.010	0.093
GFR baseline	0.989	0.975	1.003	0.123
Systolic BP baseline	1.004	0.987	1.021	0.654
Total cholesterol baseline	0.895	0.683	1.173	0.422
BMI baseline	1.079	1.007	1.157	0.031
**Heart failure / all cause death**	Hazard Ratio	[95% Conf. Lower bound	Interval] Upper bound	P-value
Intensive therapy	0.479	0.303	0.758	0.002
Age	1.087	1.046	1.130	0.000
Sex	1.068	0.626	1.822	0.809
Ejection fraction baseline	0.975	0.948	1.002	0.069
NT-proBNP baseline	1.001	1.000	1.002	0.034
HbA_{1c} baseline	1.009	0.998	1.021	0.121
u-AER baseline	1.003	0.999	1.006	0.180
GFR baseline	0.991	0.981	1.001	0.080
Systolic BP baseline	1.002	0.990	1.014	0.769
Total cholesterol baseline	1.051	0.876	1.260	0.594
BMI baseline	1.032	0.979	1.088	0.237
GFR: Glomerular filtration rate; u-AER: urinary albumin excretion rate; BP: blood pressure				

ESM table 3: Hazard ratio for increase in urinary albumin excretion rate in the highest tertile compared with the two lower tertiles.

	Hazard Ratio	[95% Conf. Lower bound	Interval] Upper bound	P-value
**Heart failure**	1.221	0.582	2.560	0.597
**Heart failure / CV death**	1.243	0.684	2.261	0.475
**Heart failure / all cause death**	1.124	0.727	1.738	0.598
Hazard ratio for increase in urinary albumin excretion rate in the highest tertile compared with the two lower tertiles in a Cox-regression including covariates as in ESM table 2. There was no association between change in albuminuria and the primary and secondary outcomes.				

ESM Figures

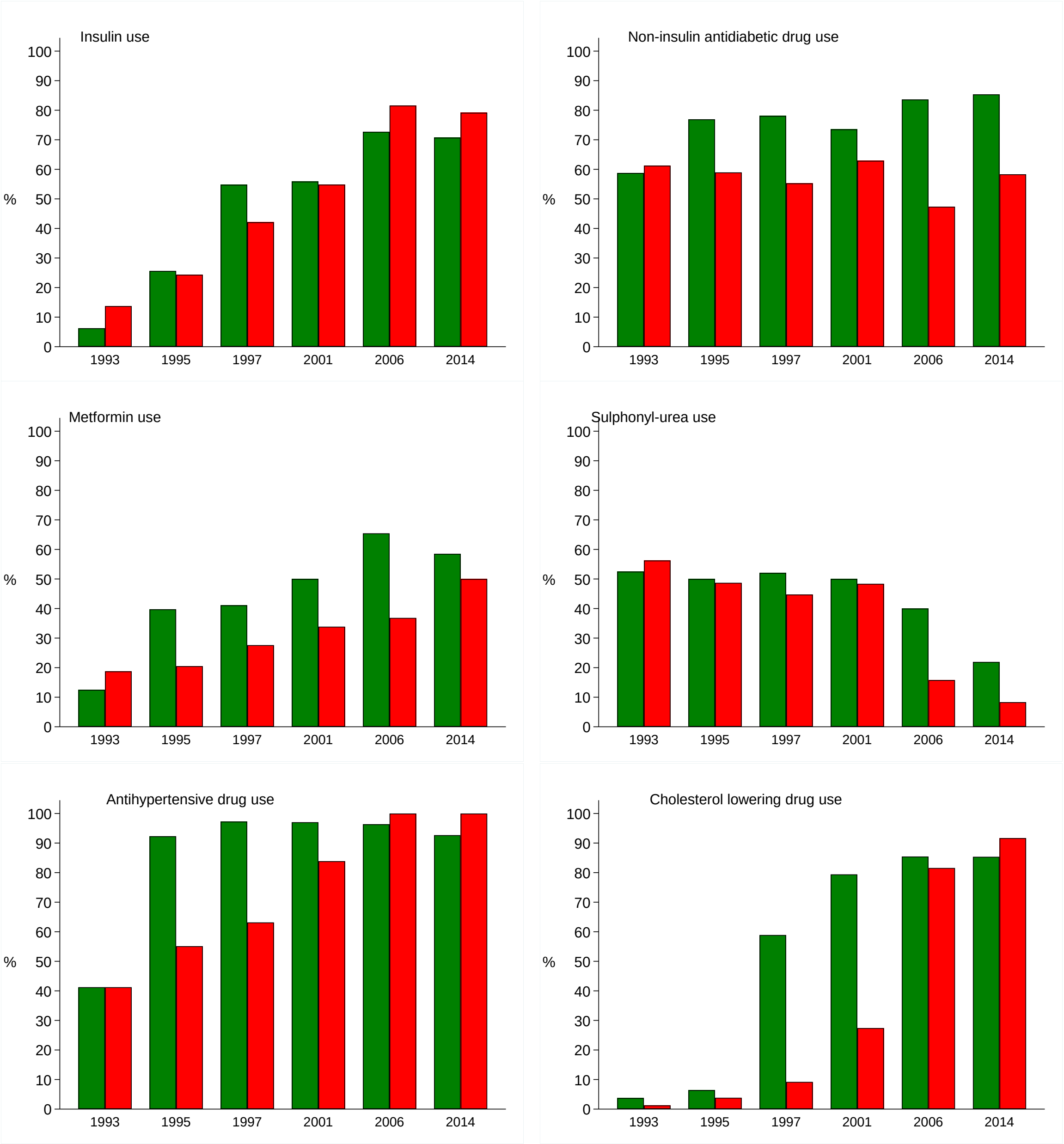

ESM figure 1: Self-reported use of insulin, other glucose lowering drugs, any antihypertensive drugs and cholesterol lowering drugs at each follow-up point. Data for medicine use between study visits was not available. Green bars, intensive-therapy group; red bars, conventional-therapy group.

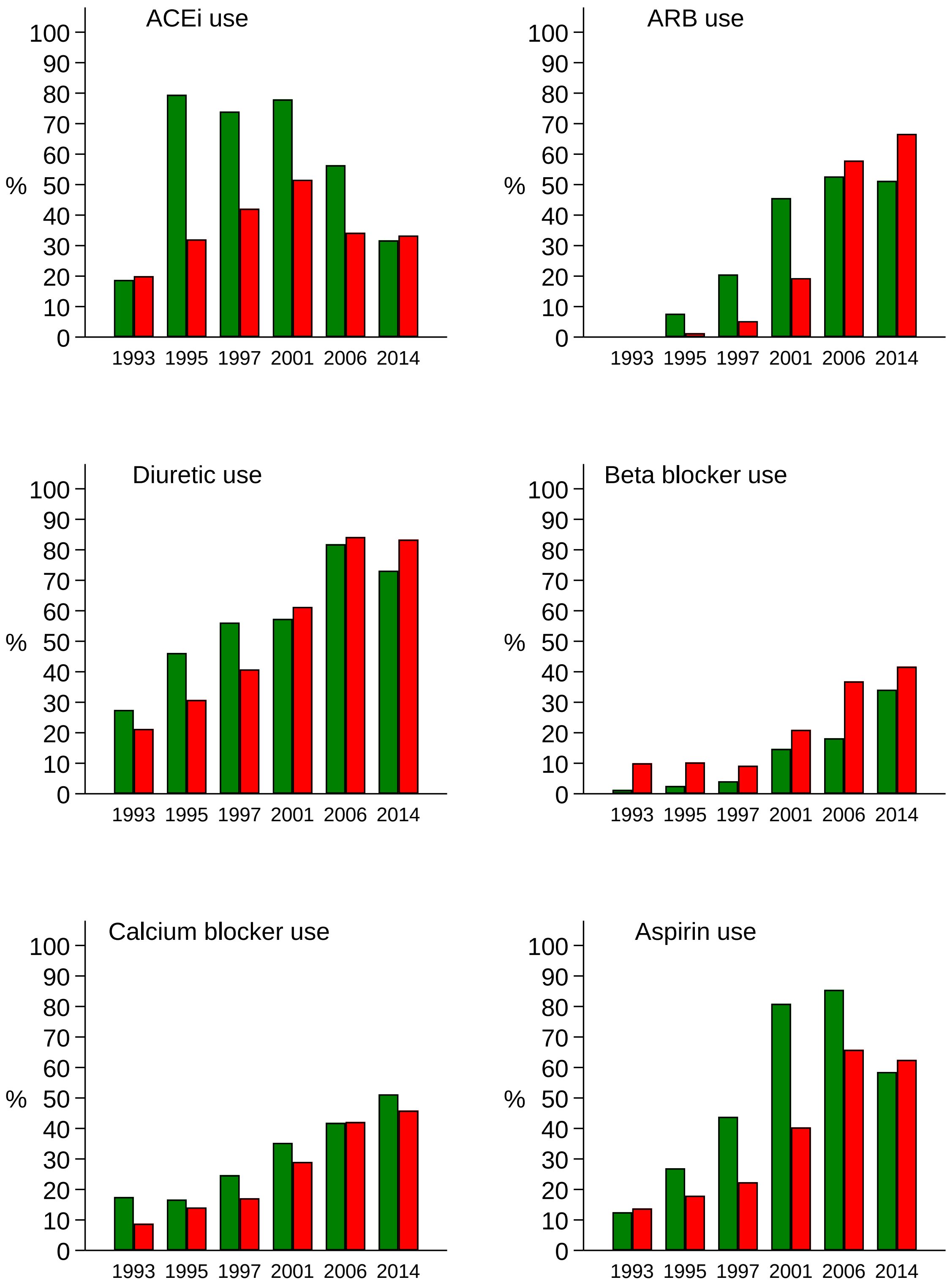

ESM figure 2: Use of specific antihypertensive drugs used. The use of other glucose lowering drugs than insulin, ACEs and ARBs as well as statins and aspirin was more widely used in the intensive-therapy group during the 7.8 years of intervention. Data for medicine use between study visits was not available. Green bars, intensive-therapy group; red bars, conventional-therapy group. ACEi: Angiotensin converting enzyme inhibitor; ARB: Angiotensin II receptor blocker.
